# Supplementary material for: Metabolic Pathway Reconstruction Indicates the Presence of Important Medicinal Compounds in Coffea Such as L-DOPA
Source: Int J Mol Sci. 2023 Aug 5;24(15):12466. doi: 10.3390/ijms241512466 (PMC10419165; doi:10.3390/ijms241512466)
Supplement: Supplementary file 1 [file ijms-24-12466-s001.zip › ijms-2499195-figures.pdf]

SuplementalDataset S1: Multiple Reaction Monitoring (MRM) chromatogram of coffee samples. The peak around the 3.7 min of acquisition time is L-DOPA.

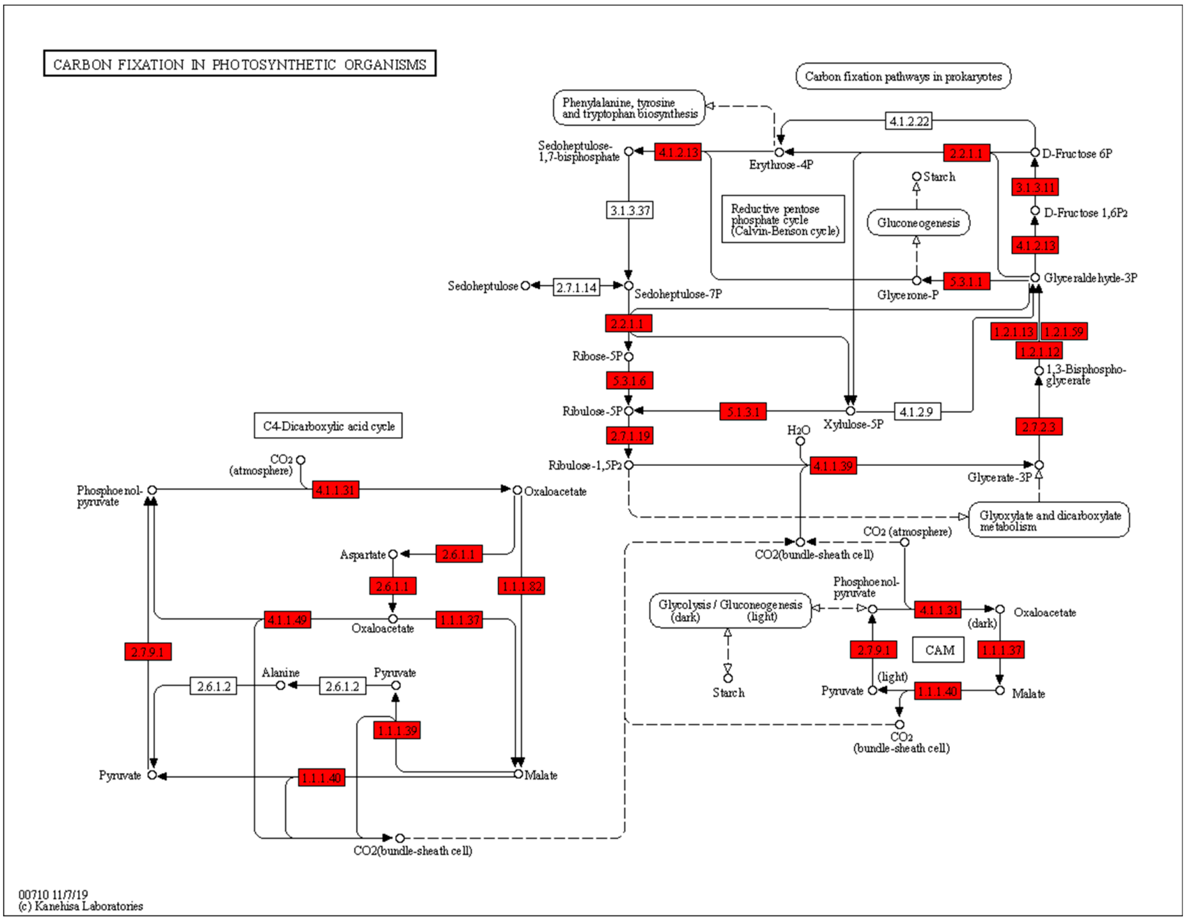

Supplemental Figure S1: The carbon fixation in photosynthetic organs map (00710 from 11/07/2019). Adapted from KEGG (Kanehisa Laboratories).

Predicted Metabolytes in Coffee leaves

Nerol	Epigallocatechin	7-Methyluric acid	Adrenosterone
Geraniol	Epcatechin	1-Methyluric acid	Testosterone glucuronide
Citronellol	Catechin	3,7-Dimethyluic acid	Phospho-ethanolamine
Coumarine	Epiafzelechin	Caffeine	Phyto-ceramide
Methylchavicol	Pinobanksin 3-acetate	3,6,8-Trimethylallantoin	Ceramide (N-Acyl-sphingosine)
Methyleugenol	5-Deoxyle ucopelargonidin	1,7-Dimethyluric acid	Lecithin
Isomethyleugenol	Dihydrofisetin	Tetradecanoic acid	3-Hexenol
Syringyl lignin	Apiforol	Hexadecanoyl-Coa	Taurine
5-Hydroxy-guaiacyl lignin	Pelargonidin	Hexadecanoic acid	5-Glutamyl-taurine
Guaiacyl lignin	Cyanidin	Octadecenoic acid	Spermine
p-Hydroxy-phenyl lignin	Delphinidin	Cholesterol	Glutathione
Caffeoyl shikinic acid	Pelargonidin 3-glucoside	Fecosterol	Thiamine
Caffeoyl-quinic acid	Cyanidin 3-glucoside	4-alpha-Methylelophenol	Nicotinate
Feruloyl-CoA	Delphinidin 3-glucoside	Cortisol	Biotin
4,2,4,6-Tetrahydroxy-3-methoxychalcone	Dopamine	Cortisone	Tetrahydrofolate
Homoeriodictyol	3,4-DHPAA	Estrone glucuronide	Retino(a)l
Luteoforol	Codeine	2-Methoxy-estradiol-17-beta-3-glucuronide	alfa-tocotrienol (Vitamin E)
Gallocatechin	Morphine	16-Glucuronide-estriol	alfa-Tocopherol
Cannabidiolate	△9-Tetrahydro-cannabionolate	Penicillin	Tetrahydrocannabinolic acid
Cannabidiol	Codeinone	(S)-Scoulerine	3-(4-Hydroxyphenyl)pyruvate

Supplemental Figure S2: Coffee leaves metabolites predicted to occur along the investigated pathways.

Retention time for L-DOPA

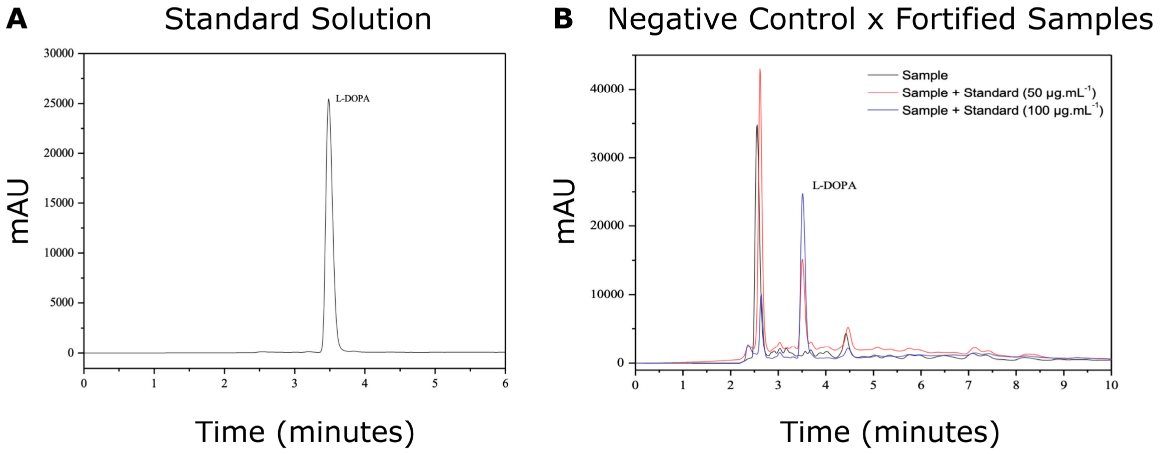

Supplemental Figure S3: Chromatogram showing the retention time for the L-DOPA standard solution. A: Retention time for L-DOPA in the standard Solution. B: Retention time for L-DOPA in the negative control fortified with L-DOPA.

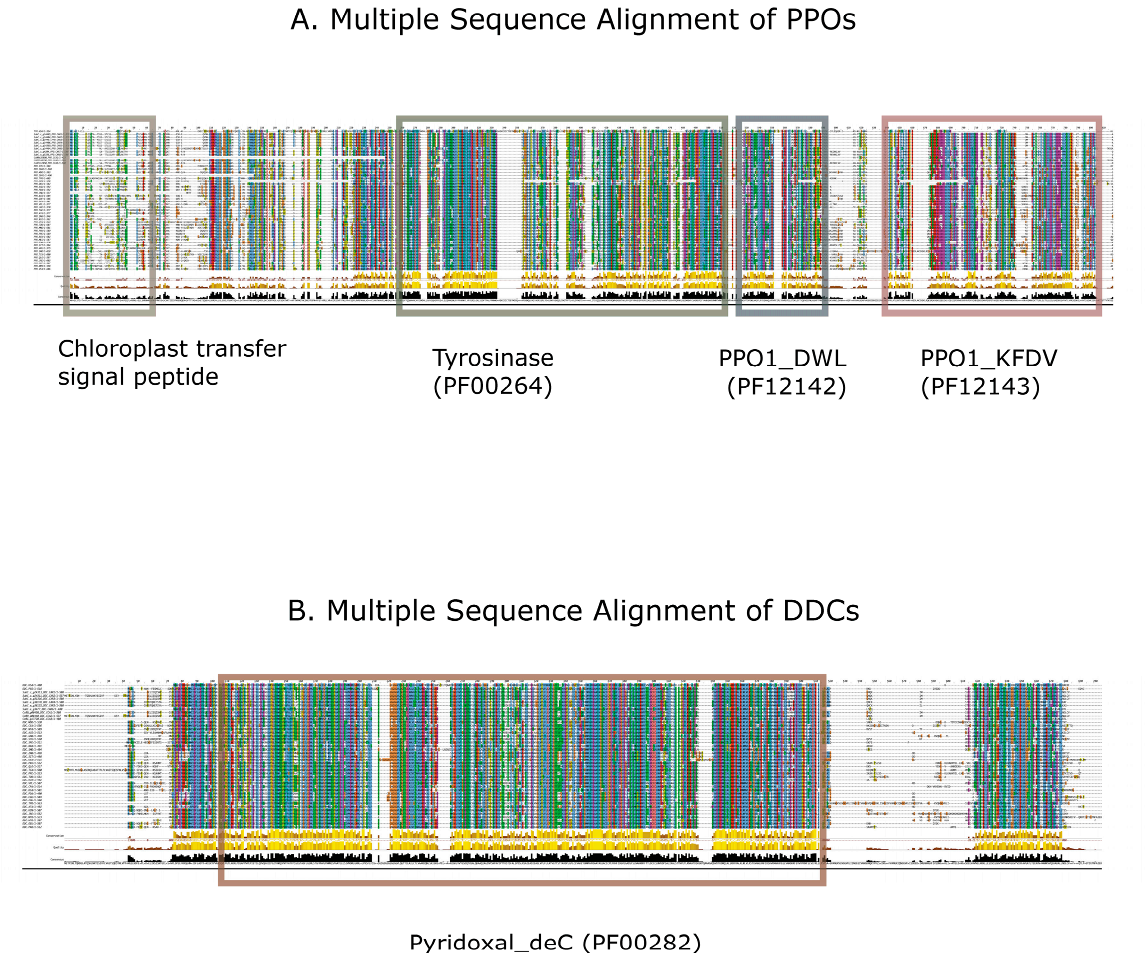

Supplemental Figure S4: Multiple Sequence Alignment of PPOs (A) and DDCs (B) used in the phylogenetic inference analysis.

Supplemental Table S1: PPO and DDCs loci in coffee plant genomes.

Supplemental Table S2: List of publicly available PPO homeolog protein sequences.

Supplemental Table S3: List of publicly available DDC homeolog protein sequences.

SupplementalDataset S2: Genomic loci of Coffee *PPO* and *DDC*.
