# Supplementary material for: Metabolic Pathway Reconstruction Indicates the Presence of Important Medicinal Compounds in Coffea Such as L-DOPA
Source: Int J Mol Sci. 2023 Aug 5;24(15):12466. doi: 10.3390/ijms241512466 (PMC10419165; doi:10.3390/ijms241512466)

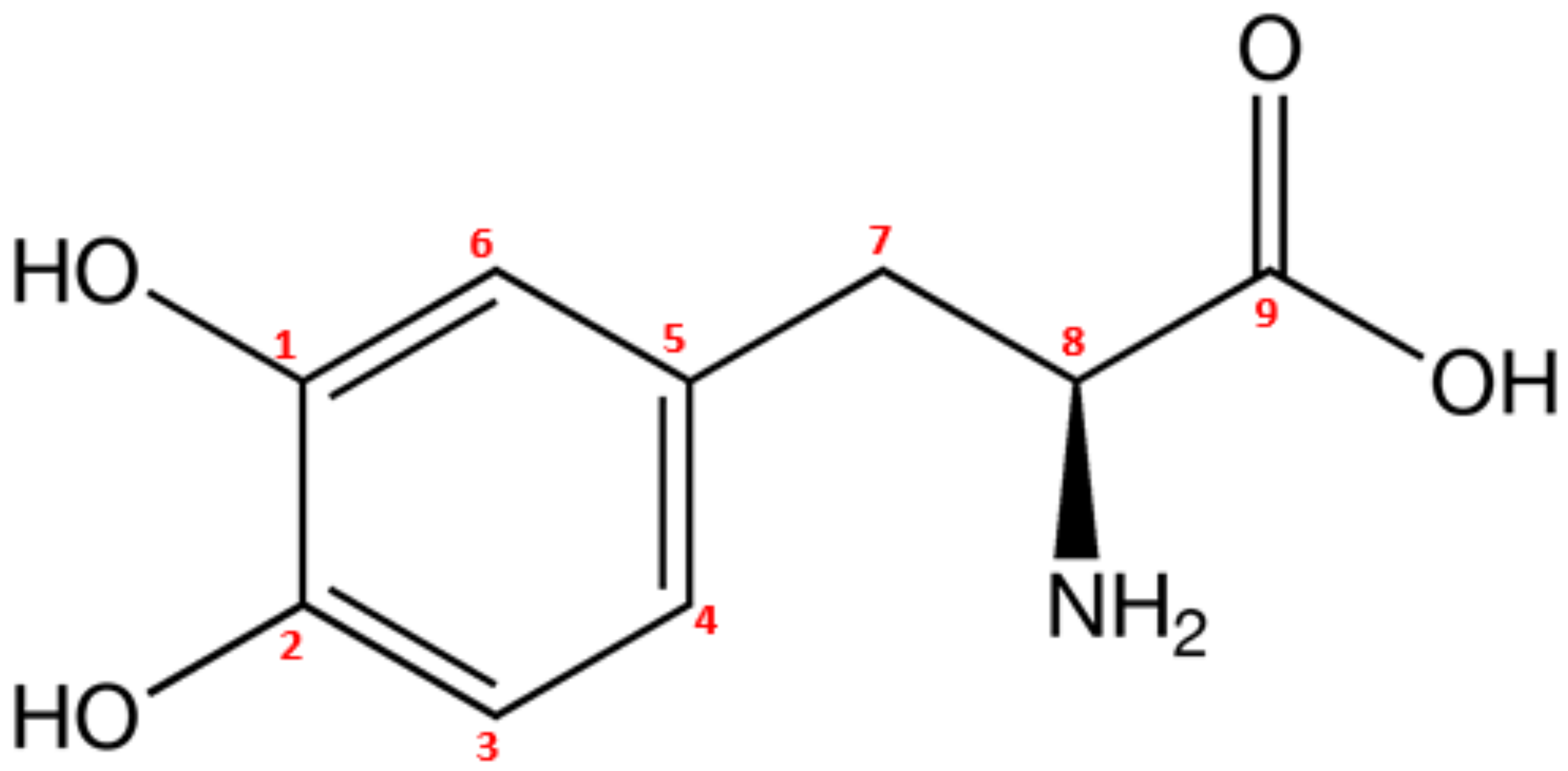


(L-3,4-dihydroxyphenylalanine; levodopa)

| Parameter | Results |
|---|---|
| Retention time ($t_r$) | 3.5 to 3.6 min |
| Linearity range* | 0.5 to 100 µg/mL (ppm) |
| Detection Limit (DL) | 0.81 µg/mL (ppm) |
| Quantification Limit (QL) | 2.73 µg/mL (ppm) |
| Correlation Coeficient ($R^2$) | 0.9999 |
| *Curve prepared in 0.1% acetic acid, from stock solution dilution. | |

| Transitions | | | Observations |
|---|---|---|---|
| m/z Precursor | m/z Product | Neutral | |
| 198 | 152 | 46 | Loss of carboxilic accid in C9 |
| 198 | 107 | 91.1 | Loss of all the ramification containing C7, C8 and C9 |
| 198 | 135 | 62.9 | Loss of both $NH_2$ in C8 and the carboxililic accid in C9, only remaining the aromatic ring with C5 linked to C7/C8 and both hydroxyls in C1 and C2. |

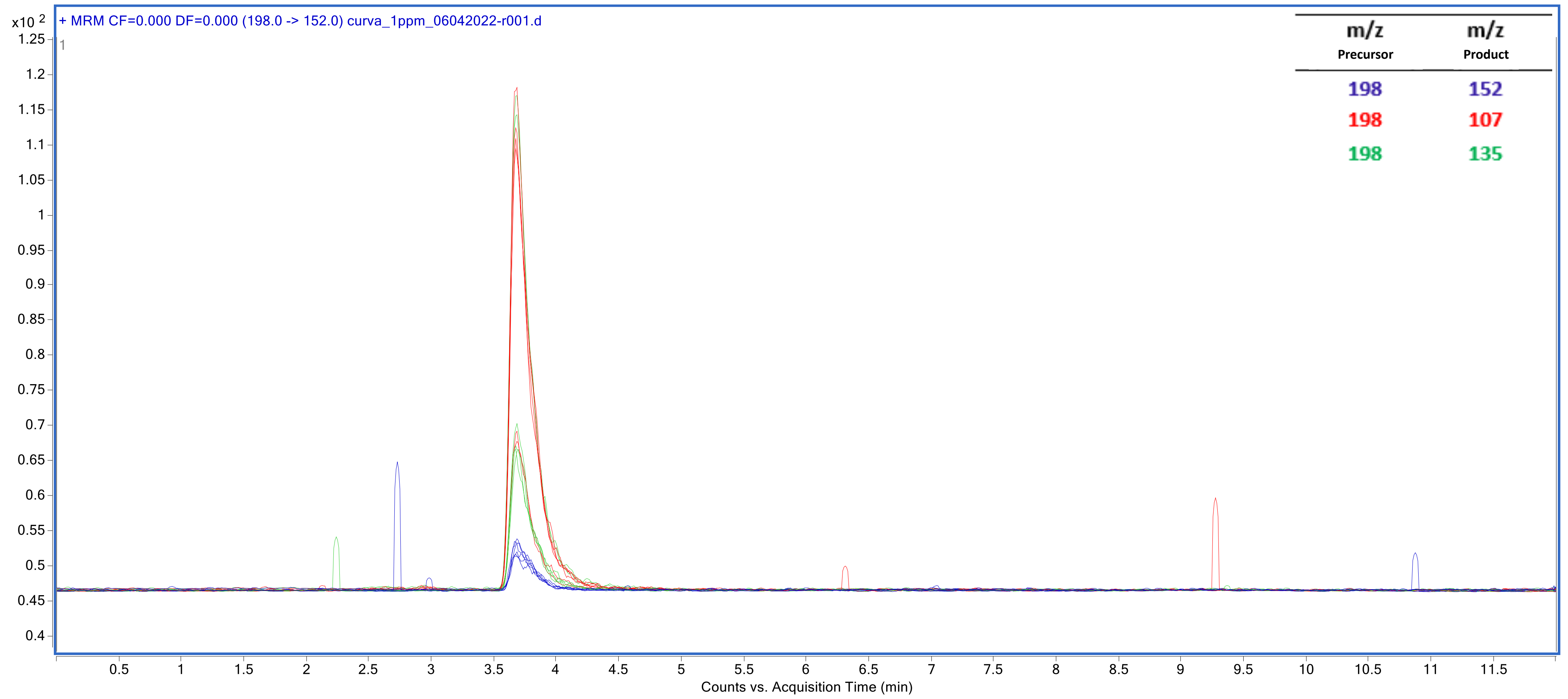


Multiple Reaction Monitoring (MRM) chromatogram whith L-DOPA standard at 1 µg/mL depicting the three characterist transitions [59–61]

## Sample 1: *C. arabica* Leaves

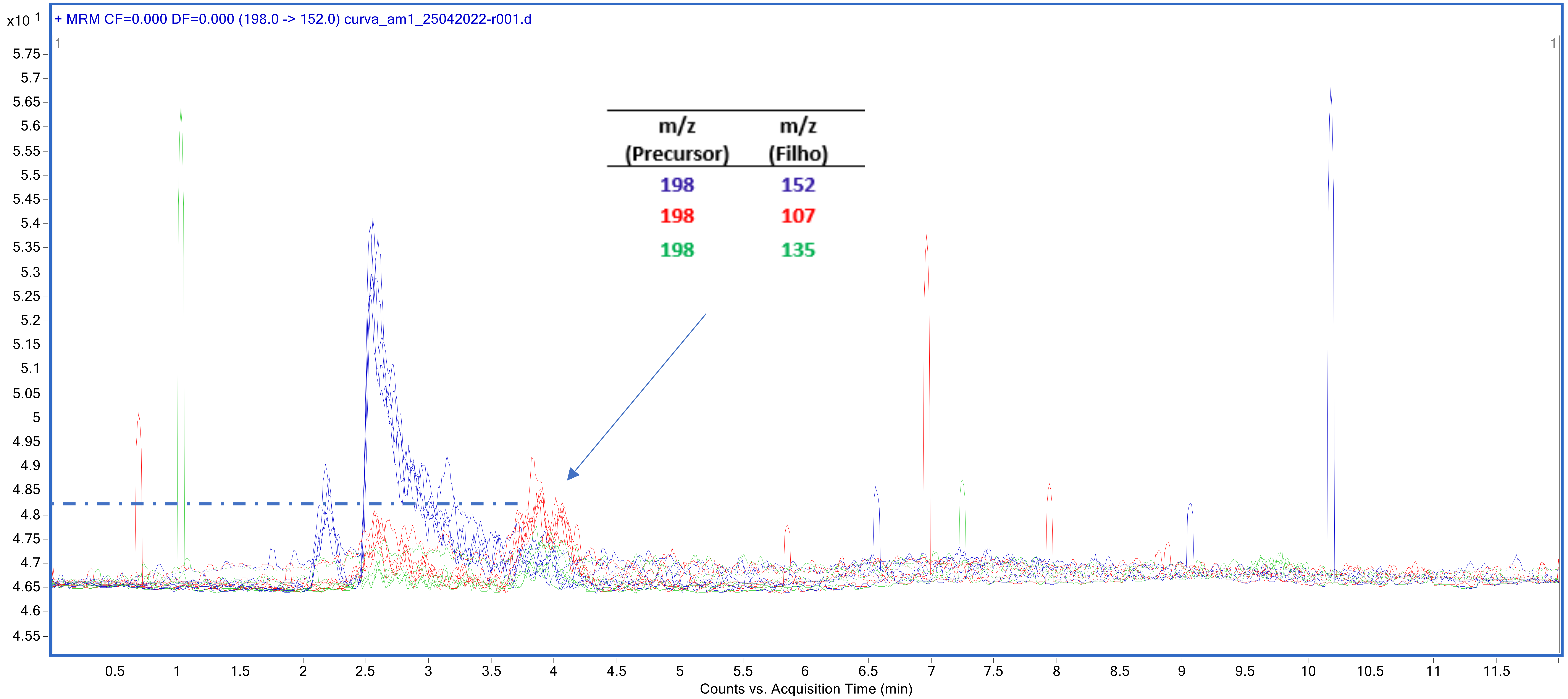

# Sample 2: *C. arabica* Leaves

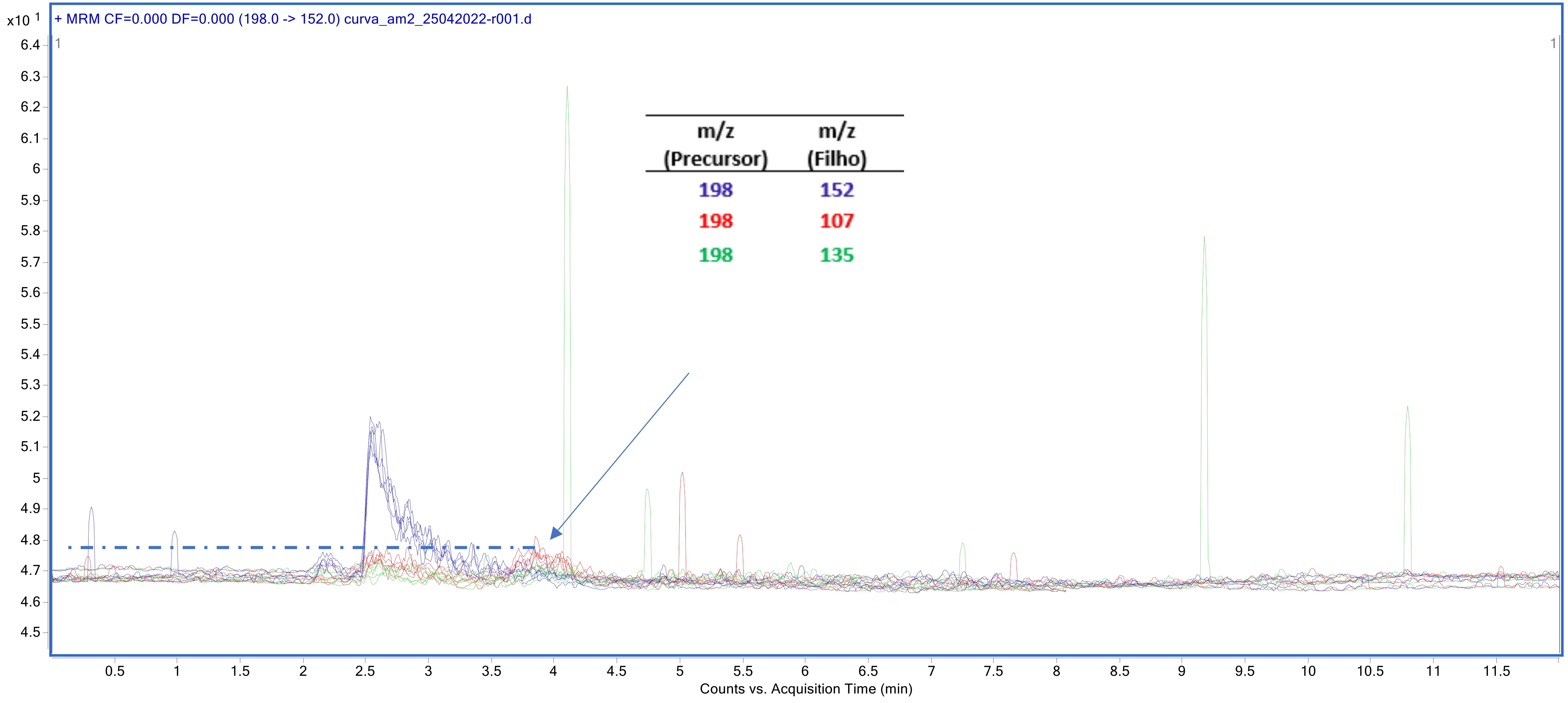

## Sample 3: *C. arabica* Leaves

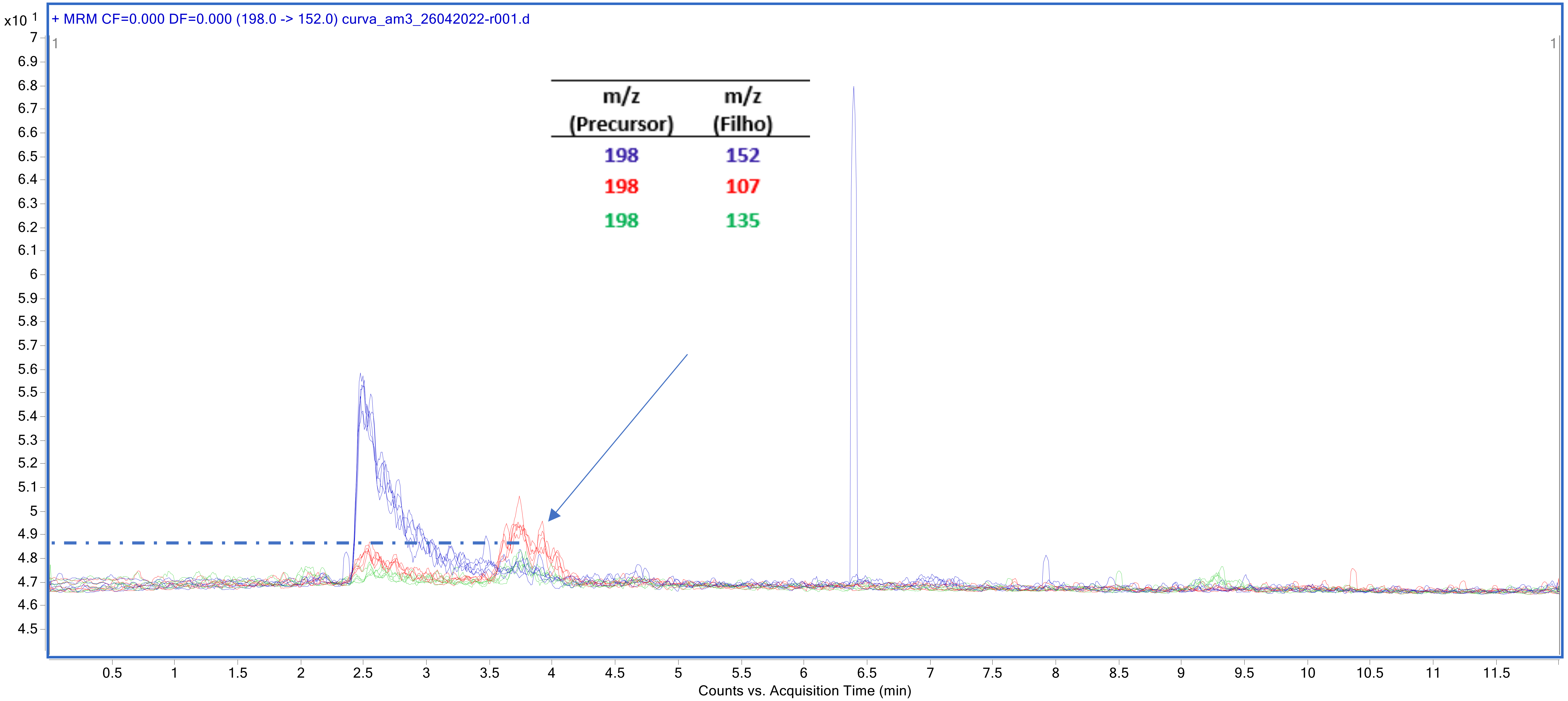

# Sample 4: *C. arabica* Flowers

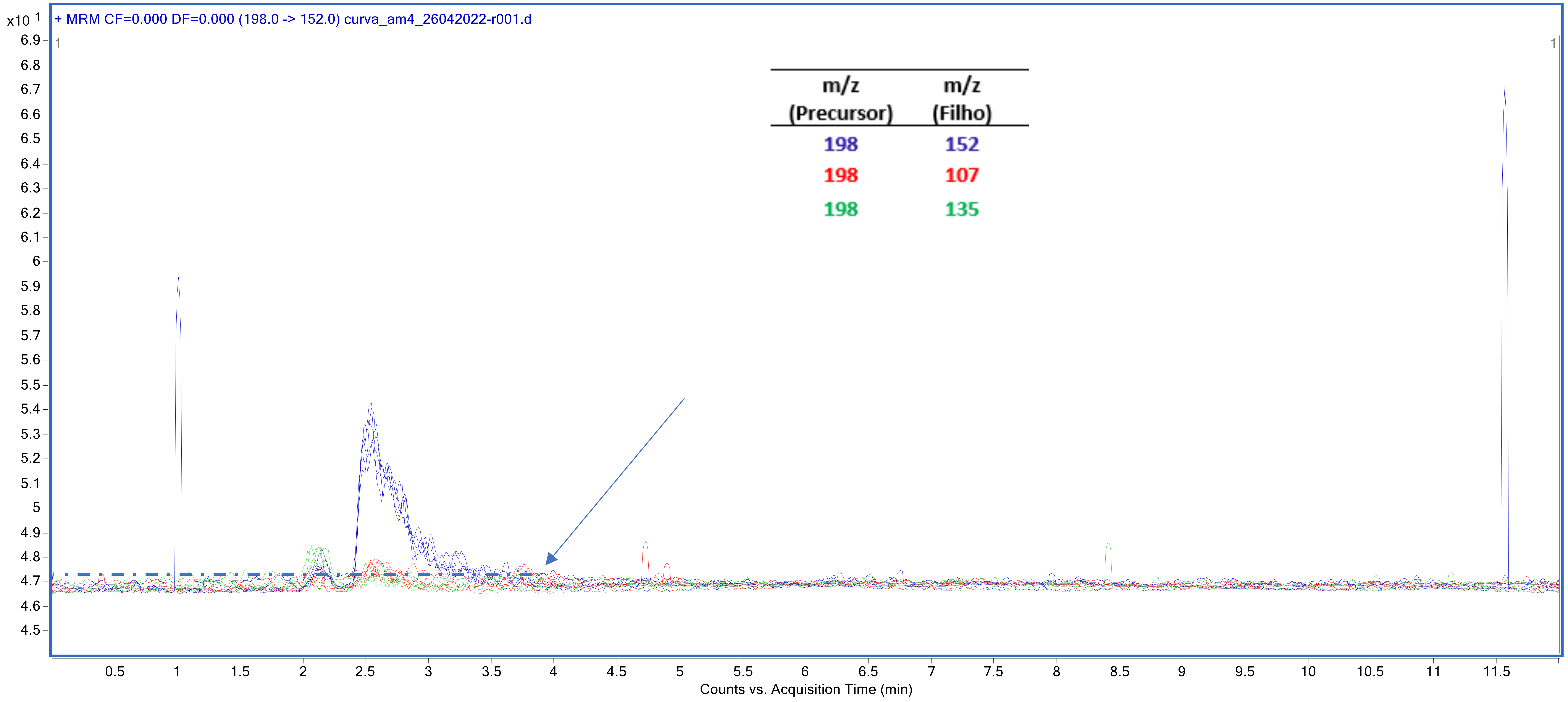

## Sample 5: *C. arabica* Flowers

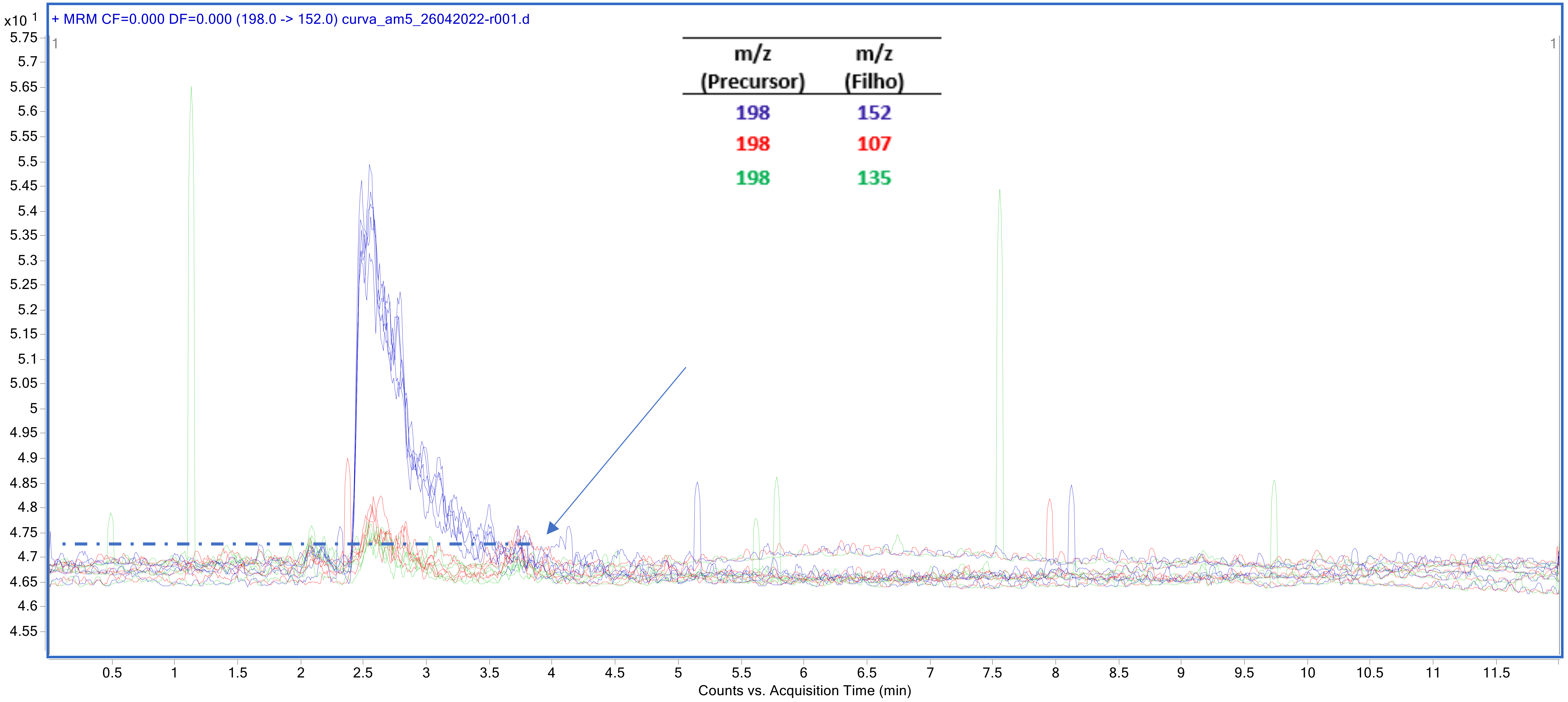


| m/z (Precursor) | m/z (Filho) |
|---|---|
| 198 | 152 |
| 198 | 107 |
| 198 | 135 |

# Sample 6: *C. arabica* Flowers

<u>Amostra 6</u>:

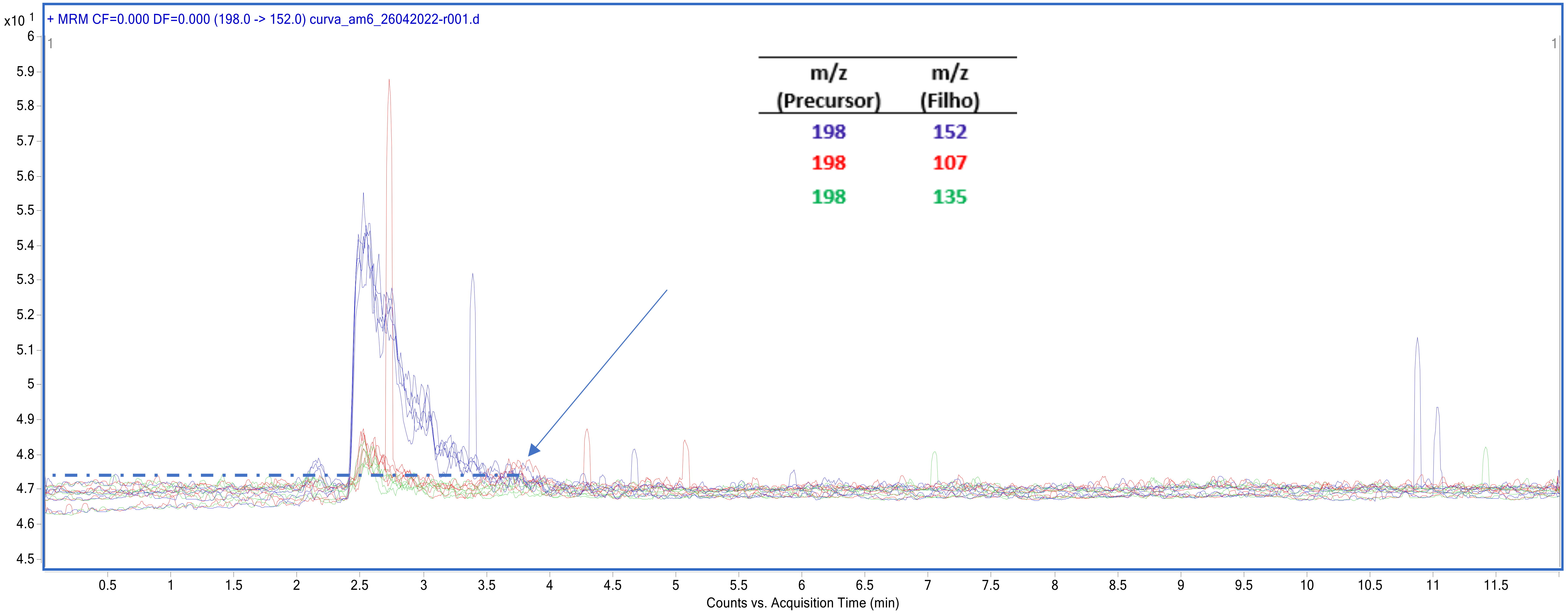


| m/z (Precursor) | m/z (Filho) |
|---|---|
| 198 | 152 |
| 198 | 107 |
| 198 | 135 |

# Sample 7: *C. arabica* Fruits

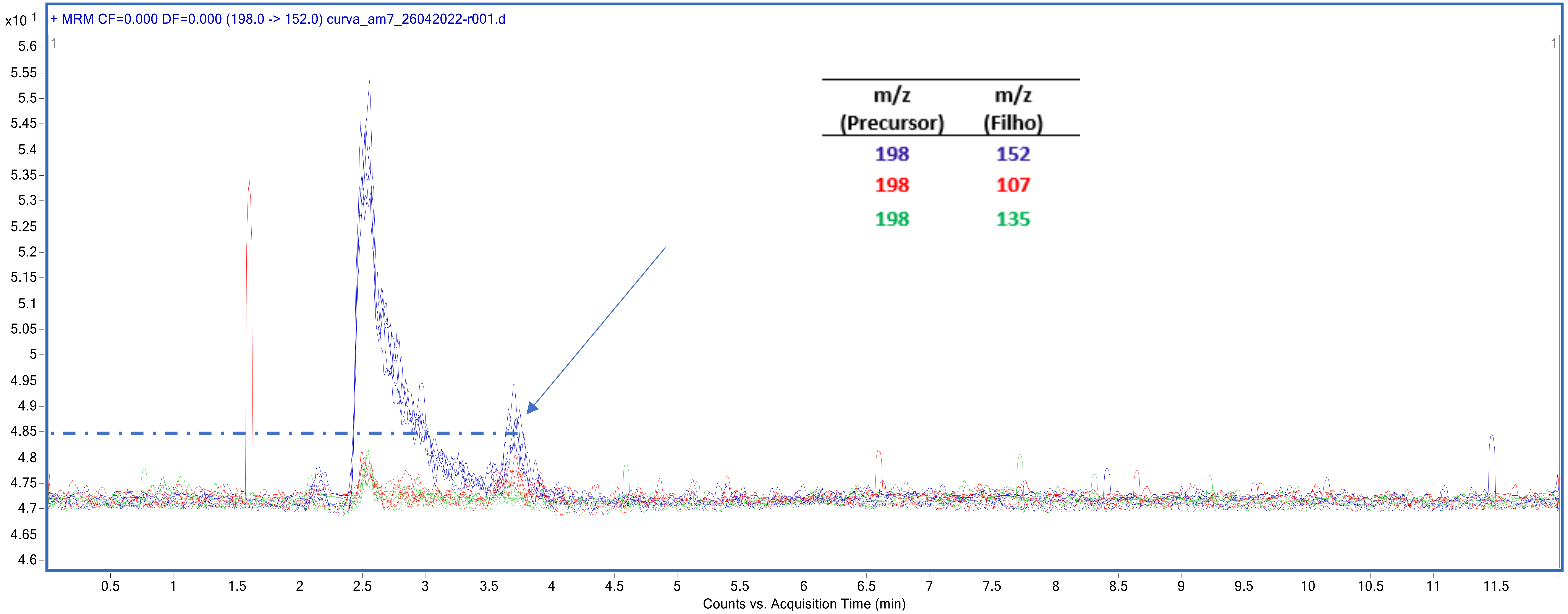

# Sample 8: *C. arabica* Fruits

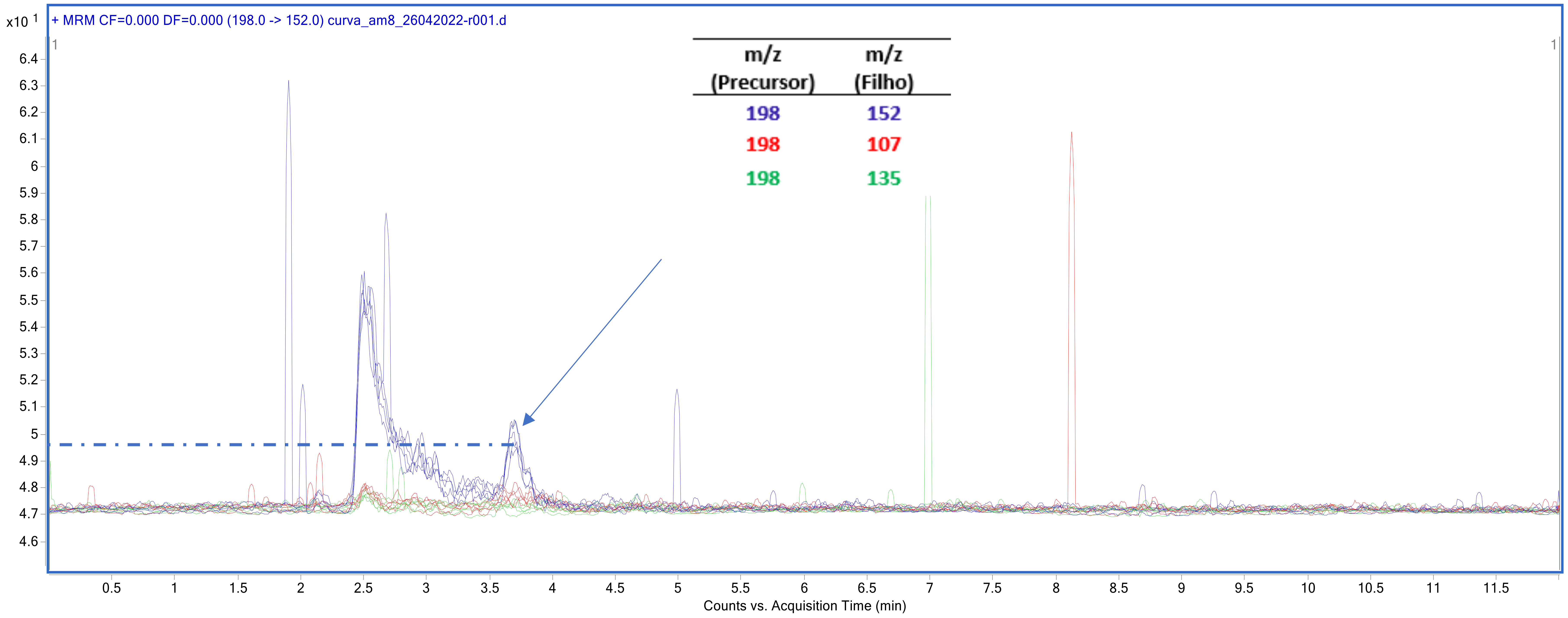

## Sample 9: *C. arabica* Fruits

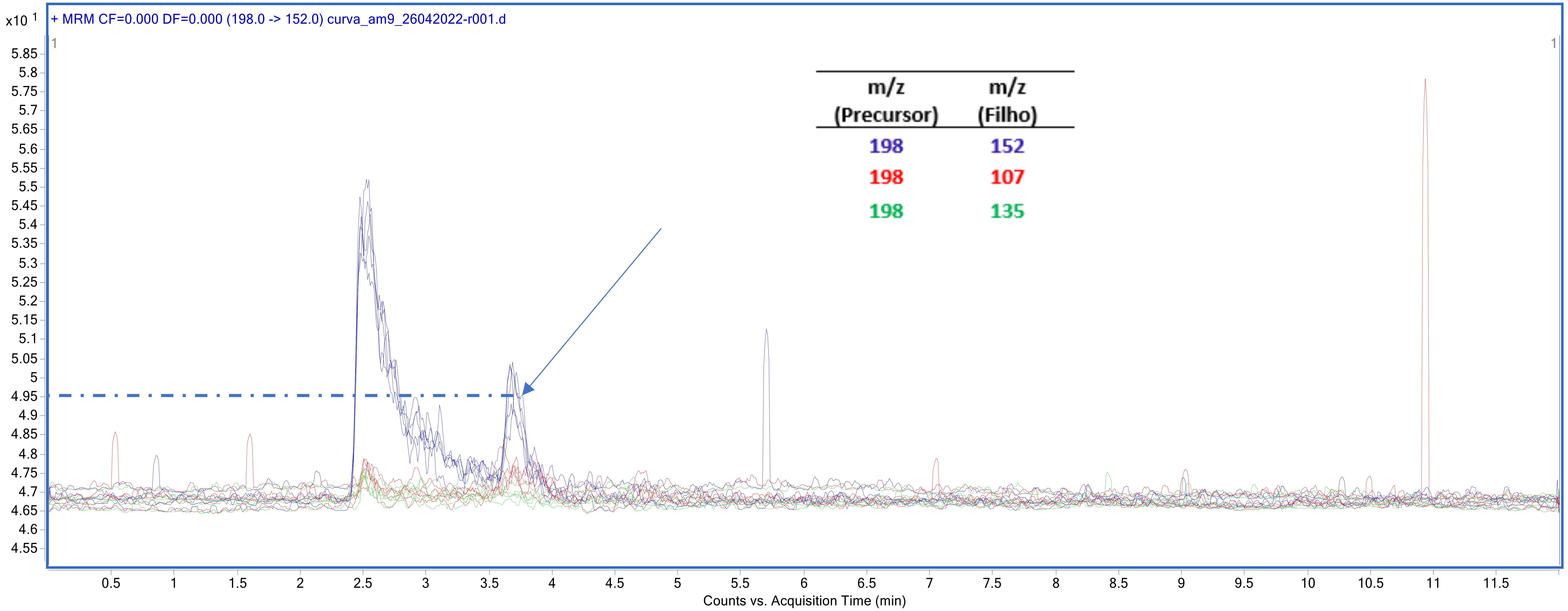

## Sample 10: *C. canephora* Leaves

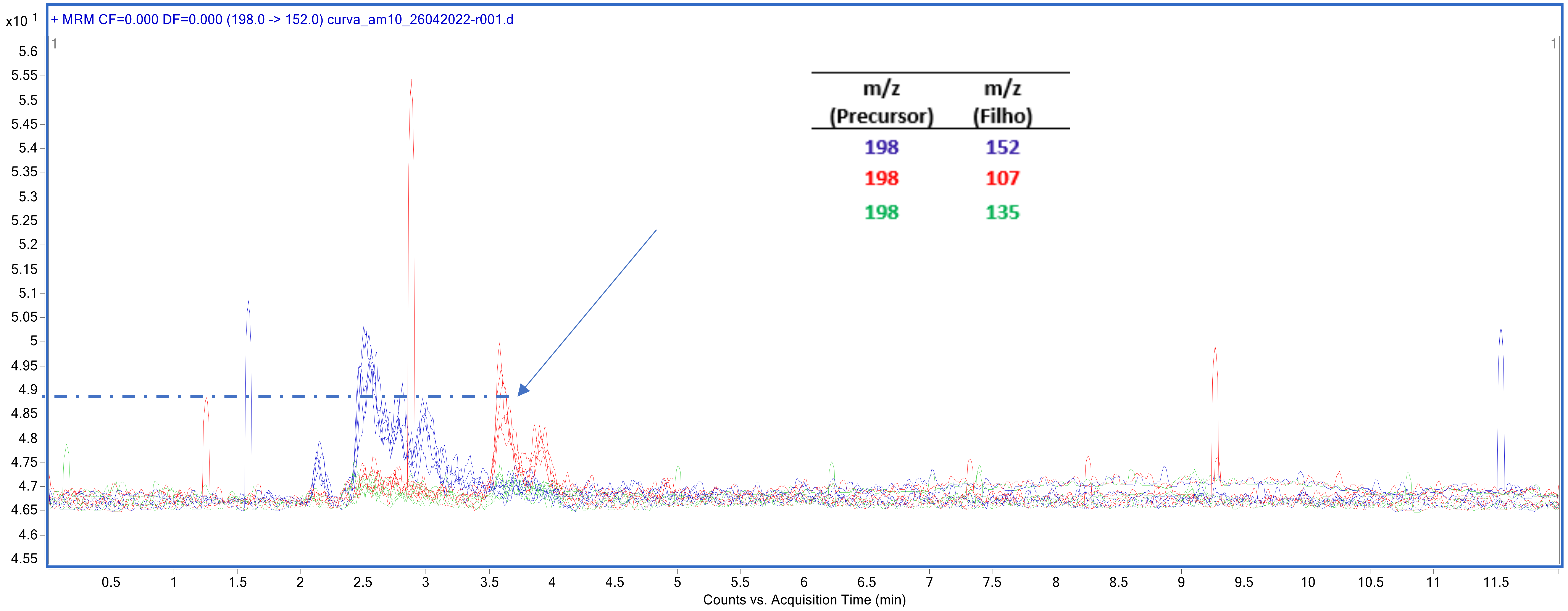

# Sample 11: *C. canephora* Leaves

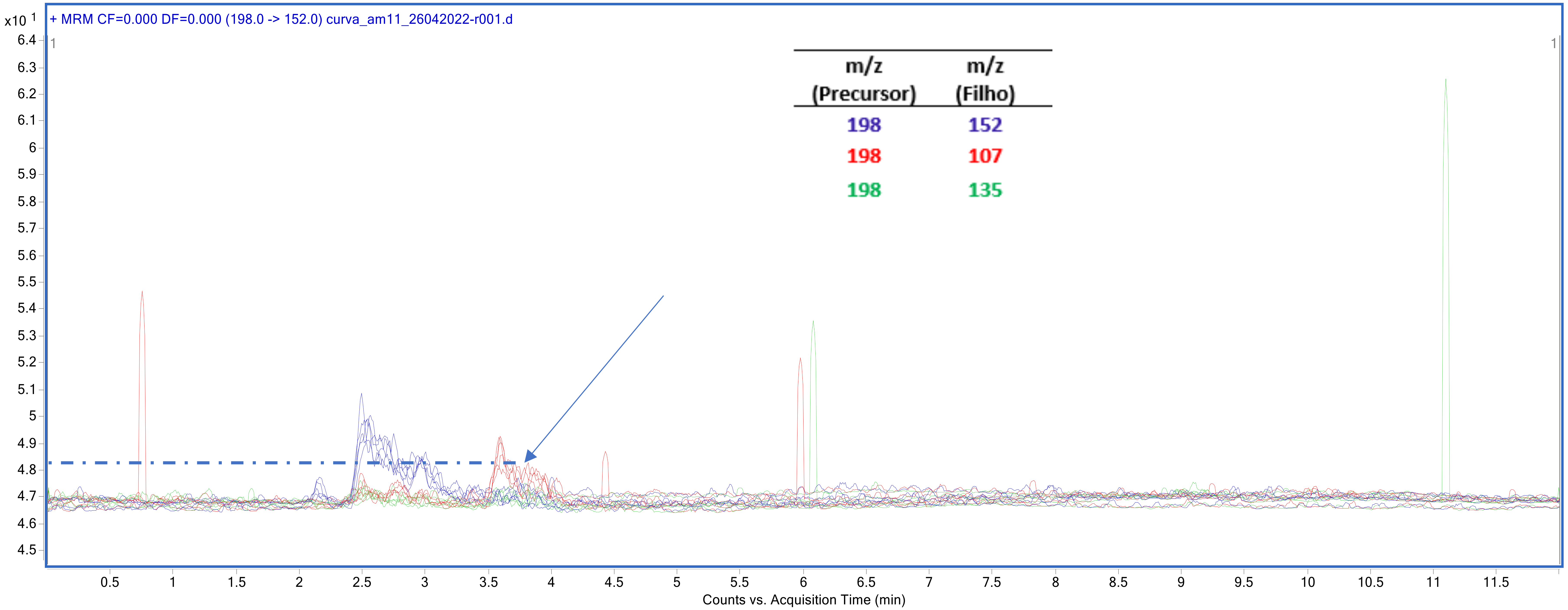

## Sample 12: *C. canephora* Leaves

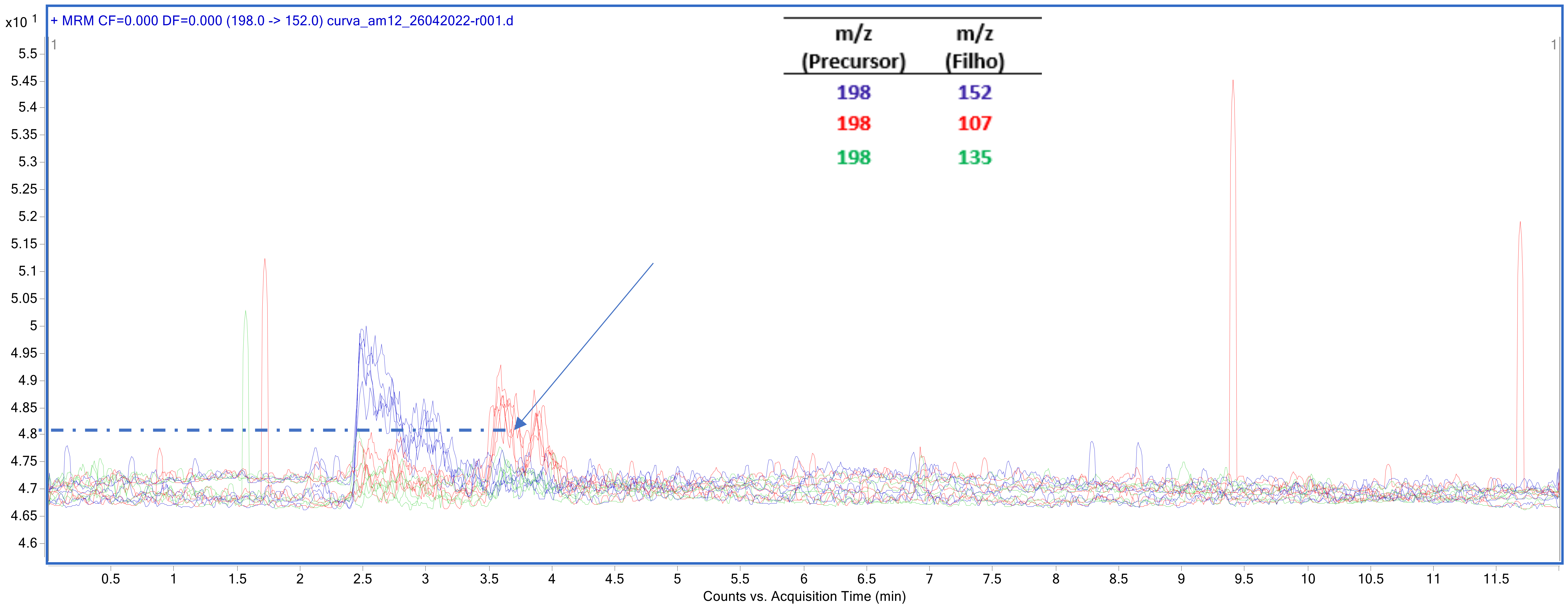

# Sample 13: *C. canephora* Flowers

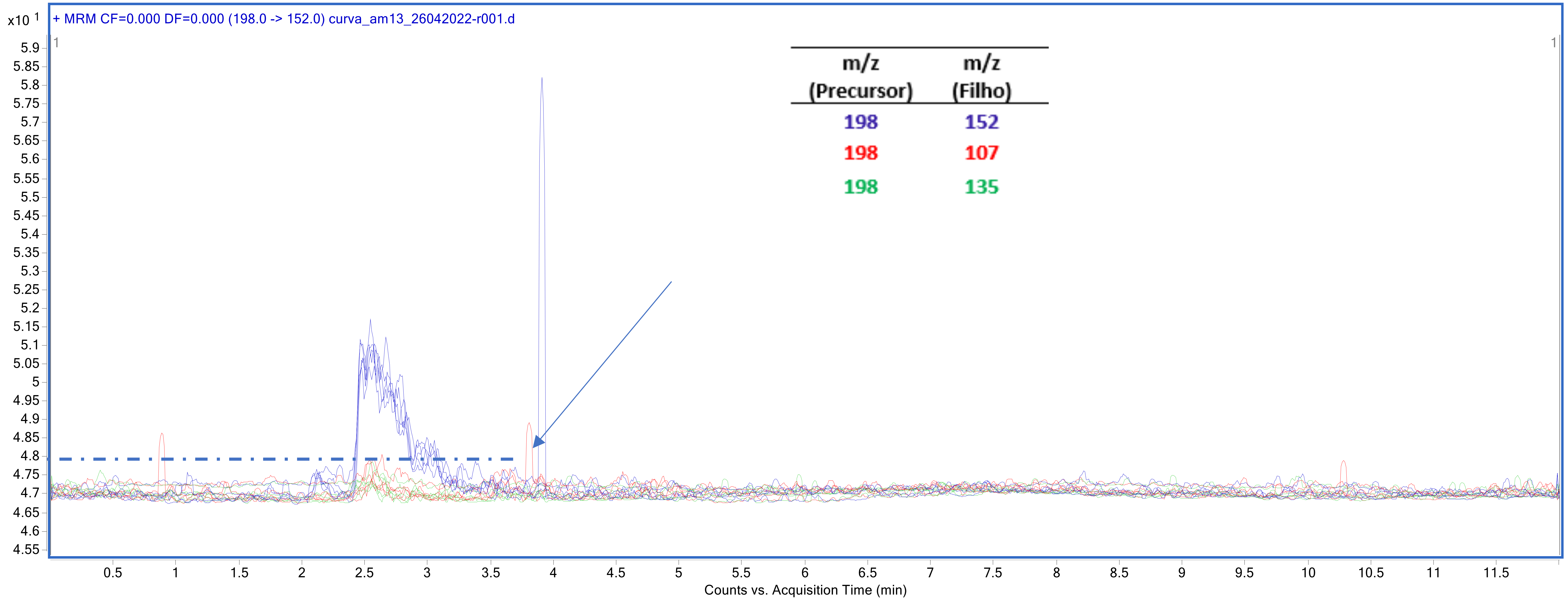

# Sample 14: *C. canephora* Flowers

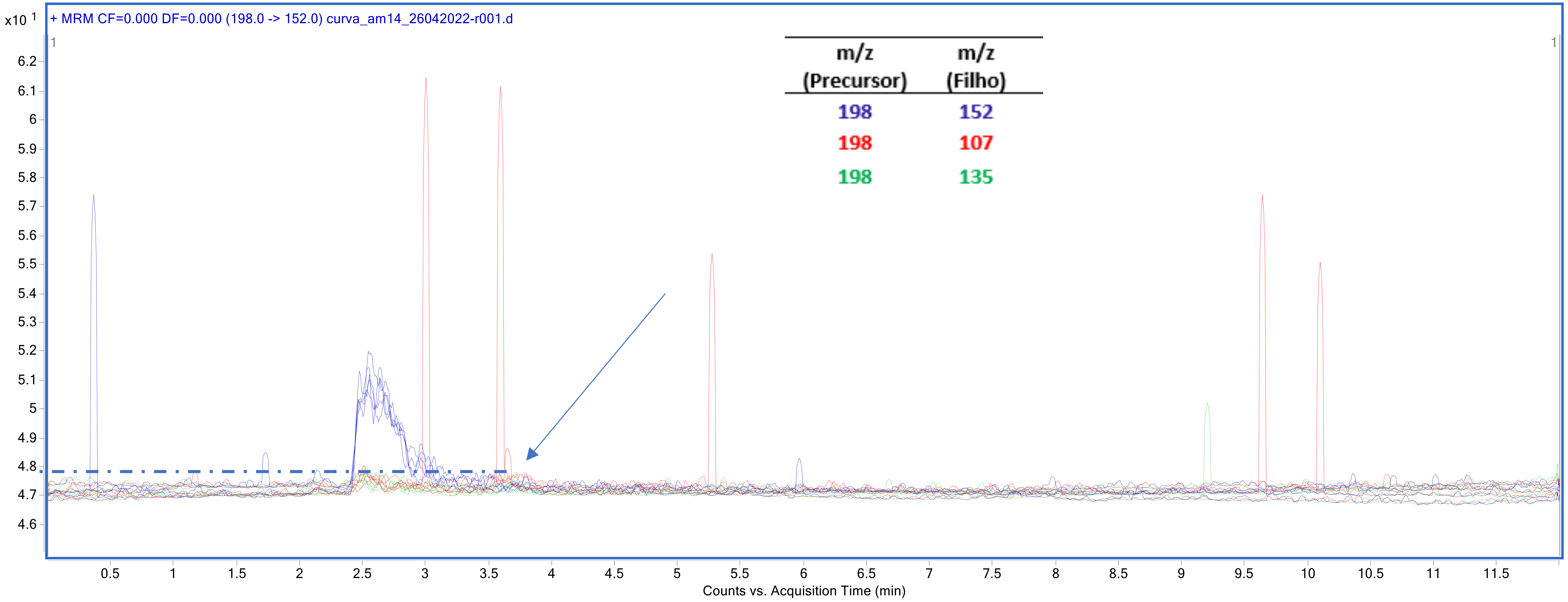

# Sample 15: *C. canephora* Flowers

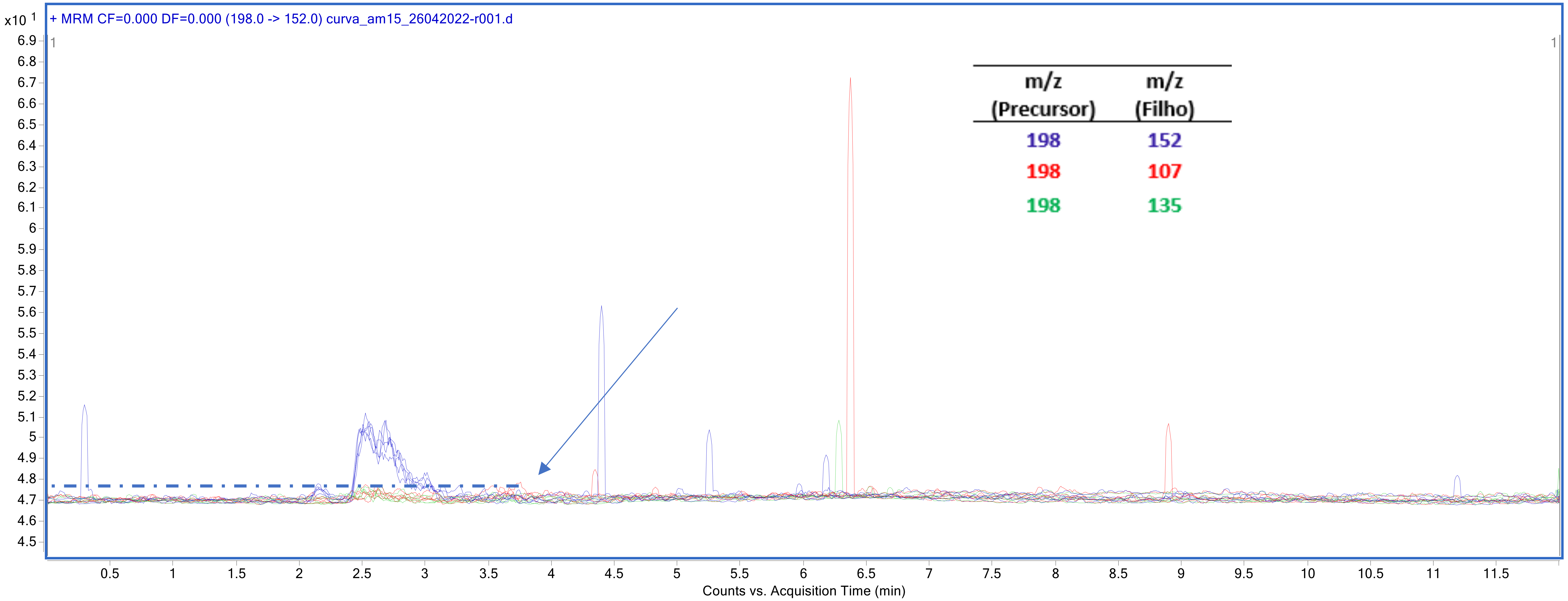


| m/z (Precursor) | m/z (Filho) |
|---|---|
| 198 | 152 |
| 198 | 107 |
| 198 | 135 |

# Sample 16: *C. canephora* Fruits

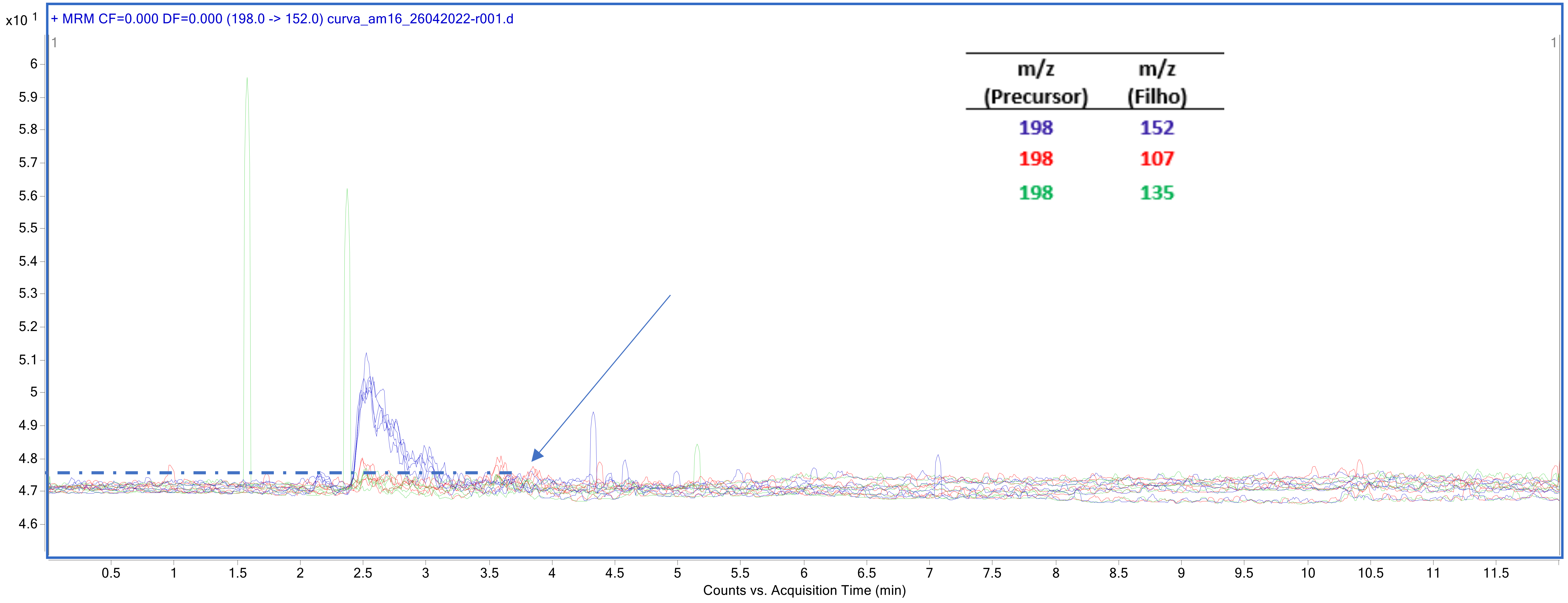


| m/z (Precursor) | m/z (Filho) |
|---|---|
| 198 | 152 |
| 198 | 107 |
| 198 | 135 |

## Sample 17: *C. canephora* Fruits

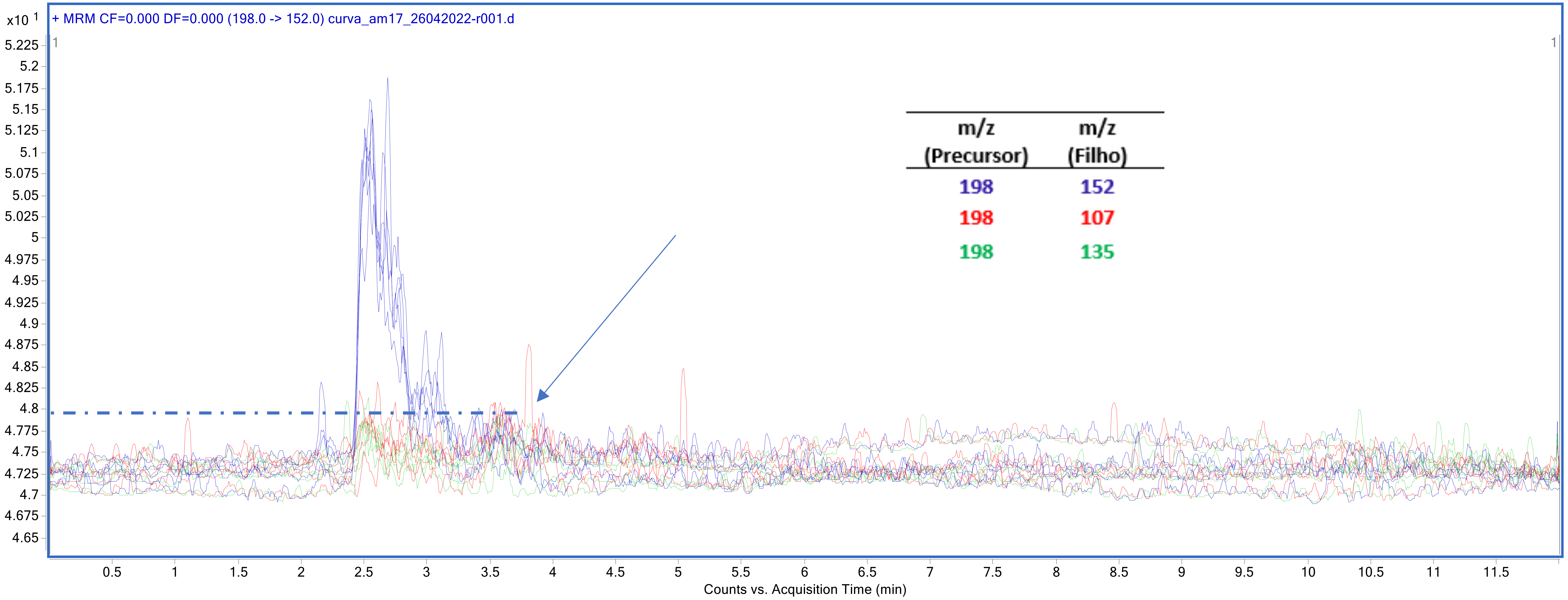

## Sample 18: *C. canephora* Fruits

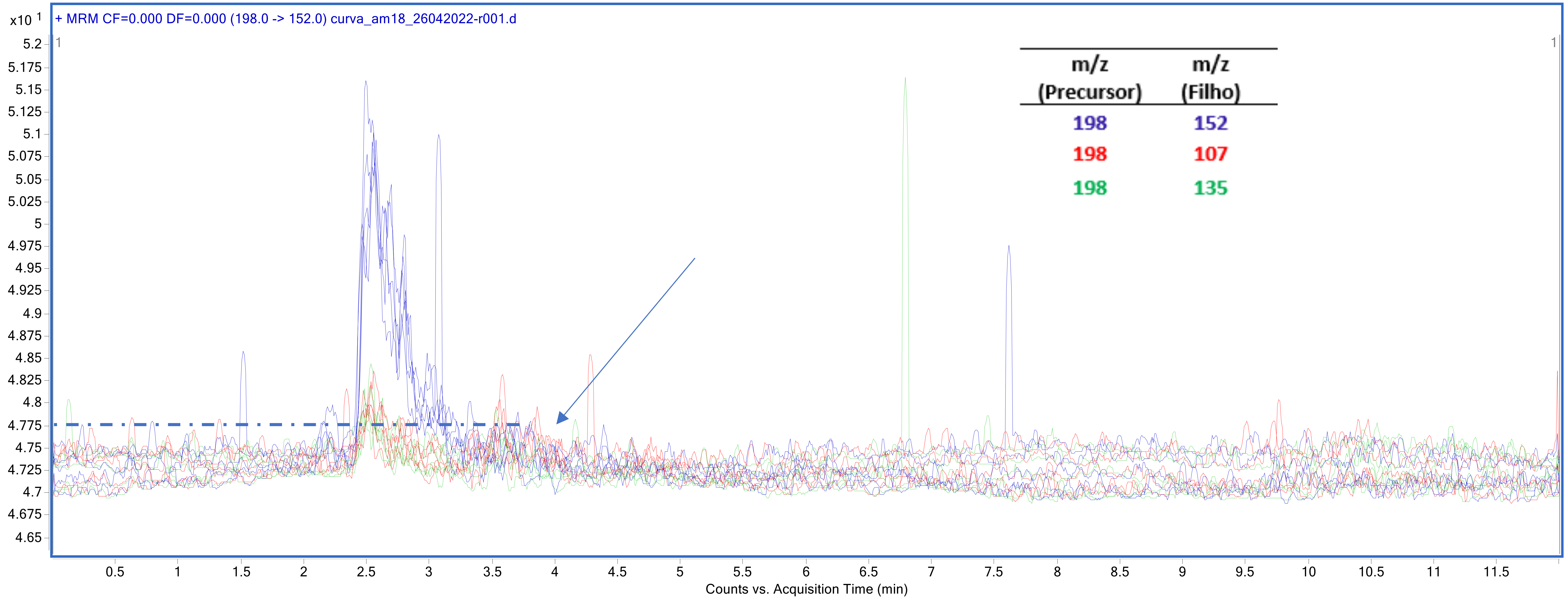

Supplement: Supplementary file 1 [file ijms-24-12466-s001.zip › SuplementalDataset S1.pdf]
